# Female sexual dysfunctions in multiple sclerosis patients with lower urinary tract symptoms: an Italian case-control study

**DOI:** 10.1093/sexmed/qfae054

**Published:** 2024-10-01

**Authors:** Raffaele Balsamo, Felice Crocetto, Biagio Barone, Ferdinando Fusco, Davide Arcaniolo, Elisabetta Costantini, Ester Illiano, Ugo Amicuzi, Marco Torella, Raffaele Ranavolo, Carmelo Quattrone, Marco De Sio, Simone Tammaro

**Affiliations:** Department of Surgical Sciences, AORN dei Colli/Monaldi Hospital, Naples 80131, Italy; Department of Neurosciences, Reproductive Sciences and Odontostomatology, University of Naples Federico II, Naples 80131, Italy; Department of Surgical Sciences, AORN Sant’anna e Sebastiano, Caserta 81100, Italy; Urology Unit, Department of Woman, Child and General and Specialized Surgery, University of Campania Luigi Vanvitelli, Naples 80131, Italy; Urology Unit, Department of Woman, Child and General and Specialized Surgery, University of Campania Luigi Vanvitelli, Naples 80131, Italy; Andrology and Urogynecological Clinic, Santa Maria Hospital Terni, University of Perugia, Perugia 05100, Italy; Andrology and Urogynecological Clinic, Santa Maria Hospital Terni, University of Perugia, Perugia 05100, Italy; Department of Surgical Sciences, AORN Sant’anna e Sebastiano, Caserta 81100, Italy; Urology Unit, Department of Woman, Child and General and Specialized Surgery, University of Campania Luigi Vanvitelli, Naples 80131, Italy; Department of Surgical Sciences, AORN dei Colli/Monaldi Hospital, Naples 80131, Italy; Urology Unit, Department of Woman, Child and General and Specialized Surgery, University of Campania Luigi Vanvitelli, Naples 80131, Italy; Urology Unit, Department of Woman, Child and General and Specialized Surgery, University of Campania Luigi Vanvitelli, Naples 80131, Italy; Urology Unit, Department of Woman, Child and General and Specialized Surgery, University of Campania Luigi Vanvitelli, Naples 80131, Italy

**Keywords:** multiple sclerosis, sexual dysfunction, lower urinary tract symptoms, LUTS, lower urinary tract dysfunction, LUTD, urodynamic

## Abstract

**Background:**

Multiple sclerosis (MS) is a recurrent, autoimmune, and inflammatory demyelinating chronic disease that typically manifests in young adulthood and exerts adverse effects on sexual functions.

**Aim:**

The study evaluated the prevalence of sexual dysfunctions (SDs) and the relationship with neurological disability, depression, and lower urinary tract symptoms (LUTS) in a cohort of MS female patients, comparing these results with those of healthy women.

**Methods:**

From January 2023 to January 2024, consecutive premenopausal female patients with MS, were recruited and the examination included urinalysis, ultrasonography and a urodynamic test according to the International Continence Society standard.

**Outcomes:**

Descriptive statistics were reported as mean and standard deviation for continuous variables (analyzed by independent samples Mann-Whitney *U* test and independent samples Kruskal-Wallis test) while categorical variables were reported as frequency and percentage (analyzed by chi-square test with Fisher’s exact test).

**Results:**

Female Sexual Function Index (FSFI) total score and all FSFI subscales scores were significantly lower in patients with MS vs healthy control subjects (*P <* .001); FSFI total scores and all FSFI subscale scores were statistically significantly lower in patients with MS with an International Prostate Symptom Score ≥20 (*P <* .001) and considering a cutoff for Beck Depression Inventory–II score ≥17, depression was present in 61% (n = 47 of 77) of patients with MS and completely absent in the control group.

**Clinical Translation:**

The knowledge that SDs are a common problem in MS and in other chronic illnesses can alleviate the feeling of stigma and talking openly of sexual problems can be helpful for the patients and so the doctor-patient relationship can be reinforced.

**Strengths and Limitations:**

The sample was drawn from a single center, and larger multicenter studies that include both genders are needed to obtain strong results.

**Conclusion:**

Our findings confirm the idea of a polygenic and multifactorial etiology of female SDs in MS. Therefore, women with MS should be evaluated in terms of SDs during follow-ups.

## Introduction

Multiple sclerosis (MS) is a chronic demyelinating disease of the central nervous system that could be declined in its 4 clinical forms: relapsing remitting, secondary progressive, primary progressive, and progressive relapsing.^1^ As neurodegenerative disease, usually diagnosed in young patients, it is one of the most frequent of the central nervous system, resulting in both somatomotor and autonomic disturbances. Signs and symptoms can be different, depending on where the lesions are located. The main autonomic disorders include sweating abnormalities, lower urinary tract dysfunctions (LUTDs), and gastrointestinal symptoms.^2^ Furthermore, depression and sexual dysfunction (SD) are common in patients with MS. Depression may be due to psychological, psychosocial, and organic pathologies arising from MS-specific brain lesions.^3^ MS affects cerebral neurophysiology, bodily functions, and psychosocial relationships,^4^ and may thus result in further SDs in female patients with MS.

SDs may be defined as complex set of conditions, associated with anatomical, physiological, biological, medical, and psychological factors.^5^ Various studies show that the prevalence of SD among female patients with MS ranges between 50% and 85%^6–8^ with loss of genital sensations, diminished libido, anorgasmia, hyporgasmia, and reduction of vaginal lubrication as the most frequently sexual complaints.^9–11^ In the general population, SD and depression are considered to be strictly related due to a known bidirectional interdependence between each other.^12^ Many previous studies also suggest an interdependence of SD, depression, disease severity, and association with MS lesions in cerebral or spinal cord areas contributing to central pathways of sexual function.^13^^,^^14^ Due to this complex interplay of factors, it is crucial for clinicians to thoroughly assess and identify the impact of SDs in patients with MS. Understanding the nuanced relationship between SDs and MS-related factors could indeed provide tailored interventions aimed at improving the overall quality of life. Data on factors associated with SD occurrence are limited, especially in Italy, and it is often hard to draw practical conclusions, as the MS population is extremely heterogeneous and generally analyzed as a whole with no MS subclass differentiation.^15^

Consequently, the primary objective of this study was to answer the following question: “What is the prevalence rate of SDs, neurological disability, depression, and other demographic data in a cohort of female Italian patients with MS in comparison with a control cohort of healthy women?” Italian medical literature on this topic is nowadays still limited. The secondary objective included the analysis of the association of depression and lower urinary tract symptoms (LUTS) with the occurrence of SDs in female patients with MS. As the final objective of the study, we tried to assess the sexual quality of life among female patients with different MS patterns in the study cohort.

## Methods

We performed a monocentric observational case-control study. Consecutive premenopausal female patients with MS in remission were recruited from our neurourological department from January 2023 to January 2024 after having their first urodynamic invasive examination. All participants provided written informed consent before enrollment, and the study was conducted in accordance with regulatory standards of Good Clinical Practice and the Declaration of Helsinki (1996)^16^ and approved by the local ethics committee (no. 148/23).

### Patients’ enrolment

The inclusion criteria for the female case group were a predetermined range of age (18-40 years); diagnosis of MS in remission, according to the McDonald revised criteria^17^; and being in a stable sexual relationship (presence of the same partners for 6 or more consecutive months). The female control group included women, in the same prefixed range of age, with none of our exclusion criteria. Exclusion criteria were as follows: history of chronic disease, hysterectomy, or vaginal surgery; receiving oral or vaginal estrogenic therapy; history of antidepressant, anticonvulsant, and anxiolytic drug use; existence of a major psychiatric disease; inflammatory disease; restricted hand, knee, and hip joints; chronic alcohol consumption; poorly controlled diabetes; and hypogonadism.

### Outcomes and measurement

The primary endpoint was to evaluate the prevalence of SD, neurological disability, depression, and other demographic data in the MS female sample (case group) in comparison with healthy women (control group). The secondary endpoint included the analysis of the association between depression and LUTS with the occurrence of SDs in female patients with MS. An accurate determination of demographic and personal features of the patients (marriage, educational status, employment, etc.) was performed. PVR and urodynamic invasive testing were determined according to the International Continence Society standard.

The Female Sexual Function Index (FSFI)^18–20^ and Sexual Quality of Life Questionnaire–Female Version (SQoL-F)^21^ were used to determine the presence of sexual life disorders, while the Beck Depression Inventory–II (BDI-II)^22^ and the International Prostate Symptom Score (IPSS)^23^ were used to identify depression and storage-voiding abnormalities. A neurological assessment using the Expanded Disability Status Scale (EDSS) in patients with MS was performed by an independent neurologist Higher EDSS score means more severe neurological condition and deficiency according to Kurtzke.^24^

### Statistics

An a priori power analysis was conducted for sample size estimation based on data from the study of Gumus et al,^25^ which compared FSFI in patients with MS and healthy control subjects. The effect size in the aforementioned study was a 25% difference in total FSFI score among the 2 groups. With a significance criterion of α = 0.05 and power = 0.80, the minimum sample size needed for this effects size is 16 patients. The decision to include a larger number of participants was driven by 2 main factors. First, we acknowledged potential variations in the actual effect size due to differences in the included cohort. Thus, increasing the sample size ensured robustness in our findings. Second, the role of our center as a referral hub for patients with SDs and LUTDs in MS facilitated recruitment beyond the required number of participants, enhancing the representativeness and validity of the study.

Descriptive statistics were reported as mean ± SD for continuous variables, while categorical variables were reported as frequency and percentage. The Kolmogorov-Smirnov test^26^ was used to assess the normal distribution of data in addition to kernel density plots. Continuous variables were further analyzed utilizing the independent samples Mann–Whitney *U* test^27^ and the independent samples Kruskal-Wallis test,^28^ while categorical variables were analyzed utilizing the chi-square test with Fisher’s exact test.^29^^,^^30^ Statistical analyses were performed using IBM SPSS version 22.0. A 2-sided *P* value <.05 was considered significant.

## Results

**Figure 1:**
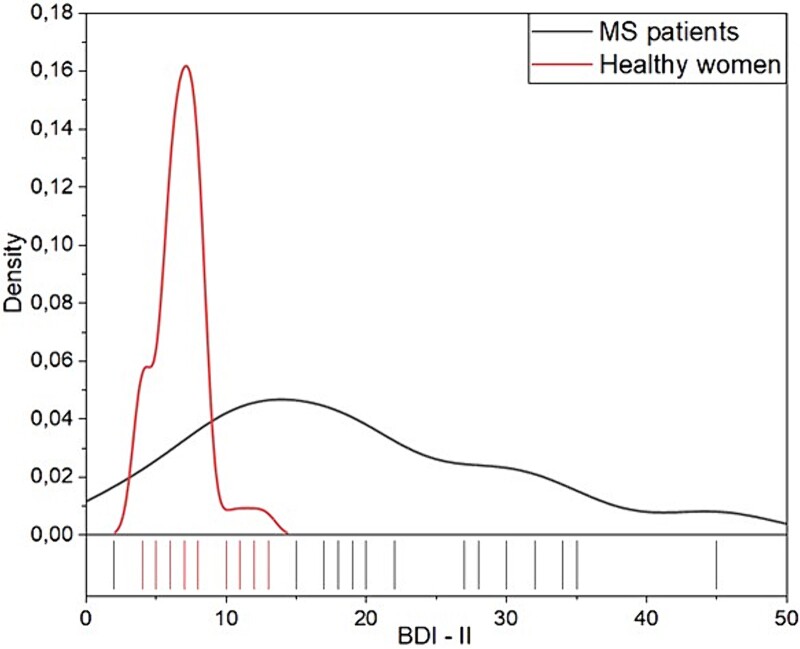
Kernel density plot for Beck Depression Inventory–II distributions.

A total of 149 patients completed this case-control study (77 cases, 72 controls). The mean age was 36.27 ± 18 for cases and 35.32 ± 5.78 for the controls. The mean duration of the disease was 13.14 ± 7.5 years. The course of MS, urodynamic findings, BDI-II, EDSS, IPSS, educational level, employment, and marital state are reported in Table 1. The mean EDSS score was 4.08 ± 1.9, with no significant difference between the types of clinical course of the disease. The mean BDI-II score was 19.00 ± 11.26 for controls and 6.81 ± 1.77 for cases (*P <* .001), while other categorial variables were similar in distribution. The kernel density plot (Figure 1) highlighted BDI-II curve density distribution for cases and controls. Considering a cutoff BDI-II depression score ≥17, depression was present in in 61% (n = 47 of 77) of patients with MS and completely absent in the control group.

**Table 1:** Demographics and neurological, urological, and sexual characteristics of patients.

**Women with MS (case group)**	**77 (100)**
Age, y	36.27 ± 6.18
Disease duration, y	13.14 ± 7.45
EDSS	4.08 ± 1.9
LUTS duration, y	4.79 ± 4.89
Day frequency	7.83 ± 2
Nocturia	1.45 ± 1.02
Urge incontinence	1.75 ± 1.91
PAD	1.65 ± 2.25
IPSS total	13.77 ± 9.3
IPSS storage	7.42 ± 5.17
IPSS voiding	6.35 ± 4.92
PVR ultrasound	64.68 ± 82.46
FSFI total	21.4 ± 4.62
FSFI desire	2.91 ± 0.94
FSFI arousal	3.31 ± 1.22
FSFI lubrication	3.74 ± 1.34
FSFI orgasm	4.05 ± 1.21
FSFI satisfaction	4.43 ± 0.98
FSFI dyspareunia	2.96 ± 0.74
BDI-II	19.00 ± 11.26
Parity	1.17 ± 0.88
MS pattern	
Relapsing remitting	59 (76.6)
Secondary progressive	18 (23.4)
Diabetes	10 (13)
Marital state	
Married	59 (76.6)
Maiden	18 (23.4)
Education	
Illiterate	11 (14.3)
Primary school (to 10 y)	10 (13.0)
High school (to 18 y)	41 (53.2)
University (>18 y)	15 (19.5)
Employment status	
Employed	49 (63.6)
Unemployed	28 (36.4)
Urodynamics	
NC	33 (42.9)
DO	31 (40.3)
DSD	2 (2.6)
DO + DSD	11 (14.3)

Values are n (%) or mean ± SD.

Abbreviations: BDI-II, Beck Depression Inventory–II; DO, detrusor overactivity; DSD, detrusor sphincter dyssynergia; EDSS, Extended Disability Status Scale; FSFI, Female Sexual Function Index; IPSS, International Prostatic Symptoms Score; LUTS, lower urinary tract symptoms; MS, multiple sclerosis; NC, normal condition; PVR, postvoiding residual.

### Prevalence of SDs

**Figure 2:**
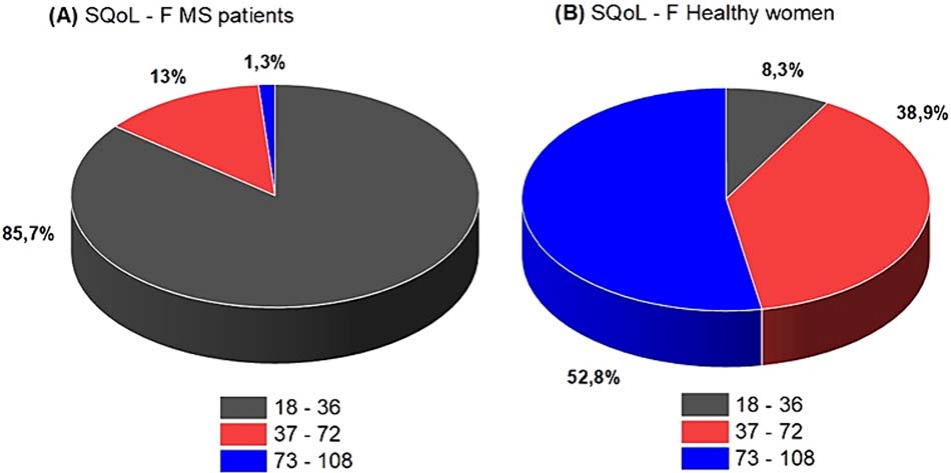
Sexual Quality of Life Questionnaire in cases and controls.

**Figure 3:**
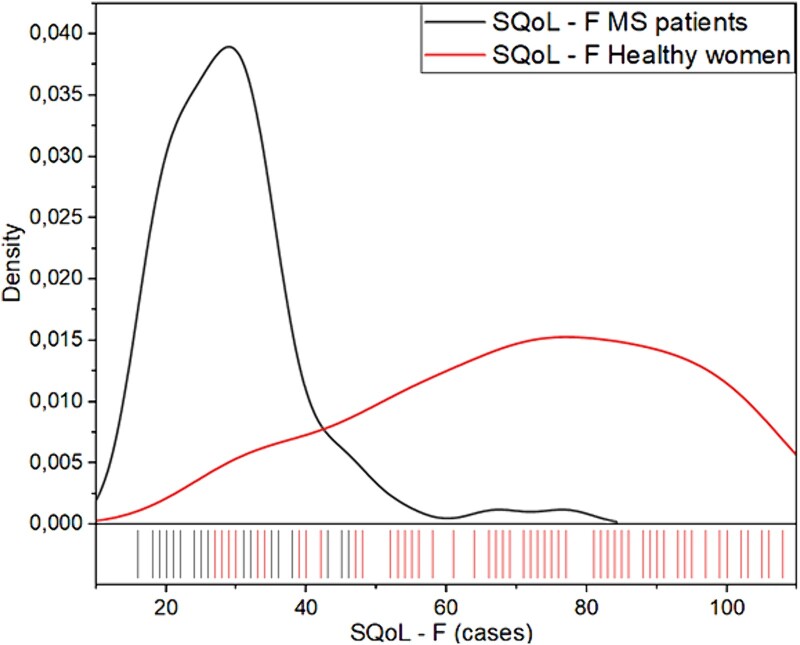
Kernel density plot for Sexual Quality of Life Questionnaire–Female Version distributions.

FSFI total scores and FSFI subscales were significantly lower in patients with MS vs healthy control subjects (*P <* .001), as shown in Table 2. There were 65 (84%) patients with MS who met the criteria for female SD (FSFI total score <26), according to the cutoff reported in the literature, while 91.67% (n = 66 of 72) of healthy women had no sexual problems. Data on sexual quality of life are reported in Table 2. The mean SQoL-F score was significantly different (*P <* .001) in patients with MS vs healthy women, 29.36 ± 10.46 for the cases vs 71.85 ± 22.23 in the control group. The pie chart (Figure 2) shows our percentage in patients with MS and healthy women, and the kernel density plot (Figure 3) highlights the SQoL-F curve density distribution for cases and controls.

**Table 2:** Characteristics of patients with MS and control subjects.

**Variables**	**Cases (n = 77)**	**Controls (n = 72)**	***P* value**
Age, y	36.27 ± 6.18	35.32 ± 5.78	.135
BDI-II	19.00 ± 11.26	6.81 ± 1.77	<.0001
Parity	1.17 ± 0.88	1.08 ± 0.71	.493
Marital state Married Maiden	59 (76.6) 18 (23.4)	58 (80.6) 14 (19.4)	.559
Education Illiterate Primary school (to 10 y) High school (to 18 y) University (>18 y)	11 (14.3) 10 (13) 41 (53.2) 15 (19.5)	9 (12.5) 10 (13.9) 38 (52.8) 15 (20.8)	.146
Employment Employed Unemployed	49 (63.6) 28 (36.4)	55 (76.4) 17 (23.6)	.09
Diabetes Yes No	10 (13) 67 (87)	11 (15.3) 61 (84.7)	.688
FSFI total FSFI desire FSFI arousal FSFI lubrication FSFI orgasm FSFI satisfaction FSFI dyspareunia	21.4 ± 4.62 2.91 ± 0.94 3.31 ± 1.22 3.74 ± 1.34 4.05 ± 1.21 4.43 ± 0.98 2.96 ± 0.74	29.81 ± 3.03 4.59 ± 0.73 5.08 ± 0.60 5.46 ± 0.53 5.36 ± 0.78 5.08 ± 1.10 4.24 ± 1.41	<.0001 <.0001 <.0001 <.0001 <.0001 <.0001 <.0001
SQoL-F SQoL-F (18-36) SQoL-F (37-72) SQoL-F (73-108)	29.39 ± 10.46 66 (85.7) 10 (13) 1 (1.3)	71.85 ± 22.23 6 (8.3) 28 (38.9) 38 (52.8)	<.0001 <.0001 <.0001 <.0001

Values are mean ± SD or n (%).

Abbreviations: BDI-II, Beck Depression Inventory–II; DO, detrusor overactivity; DSD, detrusor sphincter dyssynergia; EDSS, Extended Disability Status Scale; FSFI, Female Sexual Function Index; IPSS, International Prostatic Symptoms Score; LUTS, lower urinary tract symptoms; MS, multiple sclerosis; NC, normal condition; PVR, postvoiding residual; SQoL-F, Sexual Quality of Life Questionnaire–Female Version.

### Factors associated with SDs in female patients with MS

FSFI subscales score orgasm and dyspareunia were statistically significantly lower in patients with MS with BDI ≥17 vs patients with MS with BDI ≤17 (*P =* .029 and *P <* .0001, respectively) (Table 3). The orgasm and dyspareunia FSFI subscales were statistically significantly lower in patients with MS with EDSS ≥4.5 vs patients with MS with EDSS ≤4.5 (*P* = .025 and *P <* .0001, respectively), as shown in Table 4. FSFI total scores and scores of all FSFI subscales (sexual desire, arousal, lubrication, orgasm, satisfaction, and dyspareunia) were statistically significantly lower in patients with MS with IPSS ≥20 (severely symptomatic) as shown in Table 5.

**Table 3:** FSFI total score and subscales vs BDI-II in patients with MS.

**Domain**	**BDI-II ≤17**	**BDI-II ≥17**	***P* value**
FSFI total FSFI desire FSFI arousal FSFI lubrication FSFI orgasm FSFI satisfaction FSFI dyspareunia	22.12 ± 3.41 3.09 ± 1.01 3.67 ± 1.27 3.58 ± 1.33 4.40 ± 1.06 4.69 ± 0.82 2.77 ± 0.69	20.79 ± 5.30 2.78 ± 0.28 1.28 ± 0.46 3.93 ± 0.51 3.2 ± 0.41 4.18 ± 0.84 3.31 ± 0.18	.324 .213 .132 .296 .029 .072 .001

Values are mean ± SD.

Abbreviations: BDI-II, Beck Depression Inventory–II; FSFI, Female Sexual Function Index; MS, multiple sclerosis.

**Table 4:** FSFI total score and subscales vs EDSS in patients with MS.

**Domain**	**EDSS ≤4.5**	**EDSS ≥4.5**	***P* value**
FSFI total FSFI desire FSFI arousal FSFI lubrication FSFI orgasm FSFI satisfaction FSFI dyspareunia	21.31 ± 5.60 2.98 ± 1.06 3.44 ± 1.39 3.54 ± 1.55 4.23 ± 1.40 4.40 ± 1.11 2.70 ± 0.89	21.56 ± 3.03 2.82 ± 0.74 3.14 ± 0.93 3.98 ± 0.97 3.81 ± 0.85 4.47 ± 0.77 3.27 ± 0.23	.955 .586 .368 .140 .025 .799 <.0001

Values are mean ± SD.

Abbreviations: BDI-II, Beck Depression Inventory–II; EDSS, Expanded Disability Status Scale; MS, multiple sclerosis.

**Table 5:** FSFI total score and subscales vs IPSS total score in patients with MS.

**Domain**	**IPSS mild (0-7)**	**IPSS moderate (8-19)**	**IPSS severe (20-35)**	***P* value**
FSFI total FSFI desire FSFI arousal FSFI lubrication FSFI orgasm FSFI satisfaction FSFI dyspareunia	20.40 ± 2.69 2.60 ± 0.95 3.06 ± 1.05 3.39 ± 0.61 4.00 ± 1.05 4.29 ± 0.90 3.04 ± 0.33	24.15 ± 2.64 3.32 ± 0.81 4.03 ± 1.00 4.34 ± 1.44 4.52 ± 0.82 5.04 ± 0.48 2.90 ± 0.79	18.91 ± 6.11 2.70 ± 0.91 2.65 ± 1.16 3.32 ± 1.47 3.50 ± 1.50 3.80 ± 1.07 2.93 ± 0.74	<.0001 .003 <.0001 .001 .012 <.0001 .802

Values are mean ± SD.

Abbreviations: BDI-II, Beck Depression Inventory–II; IPSS, International Prostate Symptoms Score; MS, multiple sclerosis.

### SDs among MS pattern in female patients with MS

The FSFI total score and arousal, orgasm, satisfaction, and dyspareunia subscales were statistically significantly correlated to the MS pattern, with lower scores in secondary progressive MS as shown in Table 6.

**Table 6:** FSFI total score and subscales vs MS pattern.

**Domain**	**MS (RR)**	**MS (SP)**	***P* value**
FSFI total FSFI desire FSFI arousal FSFI lubrication FSFI orgasm FSFI satisfaction FSFI dyspareunia	21.91 ± 5.04 3.02 ± 1.04 3.54 ± 2.55 3.68 ± 1.50 4.30 ± 1.25 4.51 ± 1.01 2.85 ± 0.80	19.74 ± 2.17 2.57 ± 0.28 1.28 ± 0.46 3.93 ± 0.51 3.2 ± 0.41 4.18 ± 0.84 3.31 ± 0.18	.008 .123 .002 .893 <.0001 .033 .002

Values are mean ± SD.

Abbreviations: FSFI, Female Sexual Function Index; MS, multiple sclerosis; RR, relapsing remitting; SP, secondary progressive.

## Discussion

MS is a recurrent, autoimmune, and inflammatory demyelinating chronic disease that typically manifests in young adulthood and exerts adverse effects on sexual functions through primary, secondary, and tertiary pathways.^31^ It has been shown that the proportion of SDs in MS is greater than in other neurological diseases and is nearly 5 times more common than in the general population. Although SD is seen in approximately 40% to 80% of women with MS, the rate of inquiry into this issue remains suboptimal.^32^ In different studies, sexual functions of women with MS have been evaluated through the use of different scales, identifying among the most common symptoms dyspareunia, diminished or absent orgasm, decreased desire, decreased arousal, decreased vaginal lubrication, and reduced or absent genital sensation.^33^

Our study seeks to contribute to this underinvestigated topic, analyzing the prevalence of SDs in MS female patients, analyzing their influence on sexual quality of life, and comparing the results with those of healthy women. To our knowledge, this study represents the first Italian case-control study investigating female SD in patients with MS. According to a case-control study by Gumus et al,^25^ our series revealed a mean FSFI score of 21.4 ± 4.62 in patients with MS, while the control group exhibited a score of 29.81 ± 3.03. In an analogous study involving 1009 Turkish women, the mean FSFI score was reported to be 24.3 ± 9.5,^34^ whereas in another study, Wiegel et al^20^ reported a FSFI cutoff of 26.55. Notably, the mean FSFI score in our control subjects exceeded that of the 2 aforementioned studies, suggesting a lack of SD in our control subjects, permitting to obtain a suitable reference group for comparison with patients with MS. Consistent with the results in the literature, the present study found that the FSFI total score as well as scores of FSFI subscales (sexual desire, arousal, lubrication, orgasm, satisfaction, and dyspareunia), sexual quality of life score, and mean weekly sexual intercourse frequency were lower in women with MS compared with the control group. These data confirmed that the sexual function scores of women with MS were poorer compared with the control subjects. Potential selection bias referring to menopausal state and peripheric neuropathy in the control group was addressed by excluding individuals with these conditions from the study. Neurological disability has been associated with SDs, in particular in women affected by MS.^35^ Consistent with these findings, our study indicated that FSFI subscale scores, in particularly orgasm and dyspareunia, were statistically significantly lower in patients with MS with EDSS ≥4.5 compared with patients with MS with EDSS ≤4.5 (*P* = .025 and *P <* .0001, respectively). Additionally, our study showed a significant relationship between depression and sexual disorder, with more than a half of the patients (57.4%) meeting the criteria for depression according to the BDI-II. Notably, FSFI subscale scores for orgasm and dyspareunia were statistically significantly lower in patients with MS with BDI-II ≥17 compared with patients with MS with BDI-II ≤17 (*P* = .029 and *P* < .0001, respectively). Depression is the most common psychiatric disorder in patients with MS and is more prevalent than in other chronic diseases. In patients with MS indeed, many factors could trigger depression such as localization of brain lesions, psychosocial factors, drugs used for treatment, and patient disabilities.^36^ Consistent with other studies, the prevalence of depressive symptoms was higher in women with multiple sclerosis. Indeed, Gumus et al^25^ and Alehashemi et al^37^ found depression to be the most common mental comorbidity. A recent systematic review confirmed for the first time a bidirectional association between depression and SDs.^12^ Patients with MS who are experiencing depression might indeed not search for sexual intimacy, and conversely, patients with MS with related SDs might experience reactive depression.^12^ Our study confirms the negative correlation of female SD and sexual quality of life, as shown in Figure 1. Furthermore, at the time of the study, many patients were already undergoing antidepressant pharmacologic treatment. Thus, in an effort to avoid confounding factors, patients on antidepressant therapy were excluded from the analysis. In this study, lower FSFI subscale scores (arousal, orgasm, satisfaction, and dyspareunia) were statistically significantly correlated to secondary progressive MS, consistent with the study conducted by Confavreux et al.^38^

A high percentage (75%) of patients with MS experience LUTS. In our sample, all women with MS (100%) had experienced LUTS at least once in their lifetime and completed the IPSS. We found that the FSFI total score and FSFI subscale scores (sexual desire, arousal, lubrication, orgasm, satisfaction, and dyspareunia) were statistically significantly lower in patients with MS with IPSS ≥20 (severely symptomatic), as shown in Table 5. An association between sphincteric dysfunction and SDs was documented by Hesham et al^8^ independent of age and other comorbidities, probably due to sharing the same autonomic segment. To our knowledge, this study represents the first Italian case-control study investigating female SD in patients with MS. Nevertheless, certain limitations have to be reported. The sample was drawn from a single center, and larger multicenter studies encompassing both genders and longer follow-ups would be essential for more robust results. It is also important to acknowledge that medications administered during the course of MS treatment, such as steroids, amantadine, anticholinergics, antidepressants, and proton pump inhibitors, could affect sexual functions. Regrettably, we could not properly assess the precise impact of these drugs on our patients, and as such we excluded patients with an active phase of MS. Similarly, patients undergoing antidepressant treatment were excluded from the study, in order to avoid potential selection bias. We are conscious that this choice could have inadvertently excluded a group of patients dealing with both conditions (ie, depression and SD). Last, the self-reported nature of the data regarding IPSS and FSFI scores could have potentially lead patients involved in the study to recall bias and subjective interpretation.

## Conclusion

Our findings confirm the idea of a polygenic and multifactorial etiology of female SDs in MS. Therefore, female patients with MS should be evaluated in terms of SD both at the time of diagnosis as well as during follow-up, in order to properly assess the impact of SDs and the effects of the therapies on SDs during the course of the disease. An appropriate discussion with patients on these issues could produce some clear benefits: (1) the knowledge that SDs are a common problem in MS and in other chronic illnesses can alleviate the feeling of stigma in the patient; (2) talking openly about sexual problems can be helpful for patients; (3) the doctor-patient relationship can be reinforced; and (4) patients are more likely to be referred to or independently seek professional help that could result in improvement in their sexual functioning.
